# Supplementary material for: The Small RNA Universe of Capitella teleta
Source: Front Mol Biosci. 2022 Feb 25;9:802814. doi: 10.3389/fmolb.2022.802814 (PMC8915122; doi:10.3389/fmolb.2022.802814)
Supplement: Supplementary file 1 [file DataSheet1.ZIP › Supplement/known/CAPTEscaffold_174_10479.pdf]

Provisional ID : CAPTEscaffold_174_10479
Score total : 261761.6
Score for star read(s) : 3.9
Score for read counts : 261752
Score for mfe : 1.1
Score for randfold : 1.6
Score for cons. seed : 3
Total read count : 513427
Mature read count : 513417
Loop read count : 0
Star read count : 10

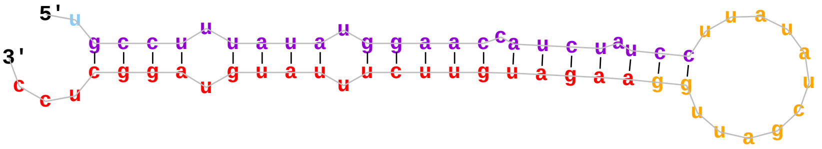

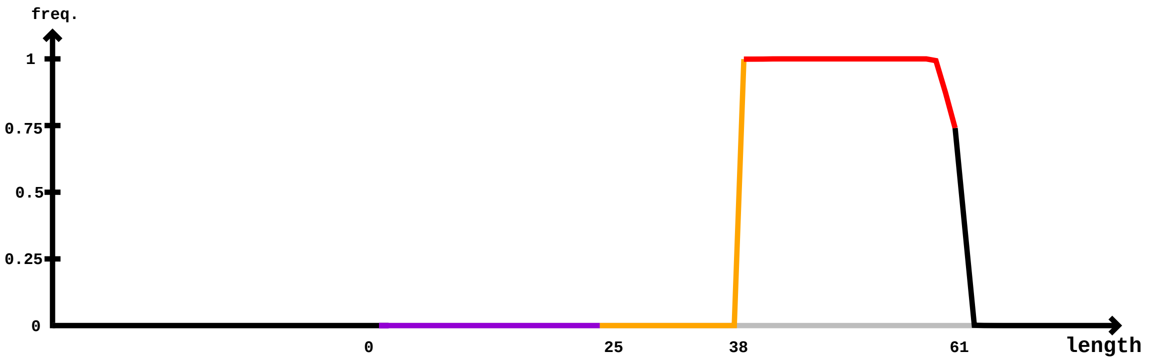


```
                                      Star                              Mature
5'- cauuaucauacauucaaaauggcggauuuuagugccuuuauauggaaccaucuauccuuauaucgauuggaagauguucuuuauguaggcuccuguucuuccgcccaau  -3'   obs
    cauuaucauacauucaaaauggcggauuuuagugccuuuauauggaaccaucuauccuuauaucgauuggaagauguucuuuauguaggcuccuguucuuccgcccaau        exp
    cauuaucauacauucaaaauggcggauuuuagugccuuuauauggaaccaucuauccuuauaucgauuggaagauguucuuuauguaggcuccuguucuuccgcccaau        known
    ....................((((((...(((.((((.((((.(((((.((((.(((..........)))))))))))).)))).))))..)))....))))))....    reads    mm    sample
    .................................gccuuuauauggaaccaucuaucc...................................................    9        0     seq
    .................................gccuuuauauggaaccaucuauccu..................................................    1        0     seq
    ...................................................................ugUaagauguucuuuauguaggcucc...............    1        1     seq
    ....................................................................ggaagauguucuuuauguaggc..................    1        0     seq
    .....................................................................Uaagauguucuuuauguaggcu.................    1        1     seq
    .....................................................................Caagauguucuuuauguaggcu.................    10       1     seq
    .....................................................................Caagauguucuuuauguaggcuc................    1        1     seq
    .....................................................................Caagauguucuuuauguaggcucc...............    37       1     seq
    .....................................................................Uaagauguucuuuauguaggcucc...............    5        1     seq
    ......................................................................aagauguucuuuauguag....................    1        0     seq
    ......................................................................aagauguAcuuuauguagg...................    1        1     seq
    ......................................................................aagauguucuuuauguagg...................    45       0     seq
    ......................................................................aagauguucuCuauguaggc..................    1        1     seq
    ......................................................................aagauguucAuuauguaggc..................    1        1     seq
    ......................................................................aagauguucuuuUuguaggc..................    1        1     seq
    ......................................................................aagauguucuuuauguaggc..................    3040     0     seq
    ......................................................................aagaAguucuuuauguaggc..................    3        1     seq
    ......................................................................aagauguAcuuuauguaggc..................    1        1     seq
    ......................................................................aagauguucuuuauCuaggc..................    1        1     seq
    ......................................................................aagauguucuAuauguaggc..................    2        1     seq
    ......................................................................aagauguucuuuauguaAgc..................    1        1     seq
    ......................................................................aagUuguucuuuauguaggc..................    1        1     seq
    ......................................................................aagauguucuuuauguaggA..................    257      1     seq
    ......................................................................aagauguucuuuaugAaggc..................    1        1     seq
    ......................................................................aagauguucuuuauguaggU..................    17       1     seq
    ......................................................................aagauguucuuuaAguaggc..................    1        1     seq
    ......................................................................aagauguucuuuauguaggG..................    2        1     seq
    ......................................................................aagauguucCuuauguaggc..................    2        1     seq
    ......................................................................aagaugAucuuuauguaggc..................    2        1     seq
    ......................................................................Gagauguucuuuauguaggc..................    1        1     seq
    ......................................................................aagaugGucuuuauguaggcu.................    18       1     seq
    ......................................................................aagauguucuuuauguaggGu.................    4        1     seq
```

Star **Mature**

```
cauuaucauacauucaaaauggcggauuuuagugccuuuauauggaaccaucuauccuuauaucgauuggaagauguucuuuauguaggcuccuguucuuccgcccaau
......................................................................aagauguGcuuuauguaggcu..................  2      1  seq
......................................................................aagauguucuuuauguaggcG..................  243    1  seq
......................................................................aagauguucCuuauguaggcu..................  14     1  seq
......................................................................aagauguucNuuauguaggcu..................  1      1  seq
......................................................................aagauguucuuuauUuaggcu..................  5      1  seq
......................................................................aagauguucGuuauguaggcu..................  7      1  seq
......................................................................aNgauguucuuuauguaggcu..................  2      1  seq
......................................................................aagaCguucuuuauguaggcu..................  2      1  seq
......................................................................aagaugAucuuuauguaggcu..................  48     1  seq
......................................................................aCgauguucuuuauguaggcu..................  1      1  seq
......................................................................aagauUucuuuauguaggcu..................  3      1  seq
......................................................................aagauguucuuuauguaggUu..................  9      1  seq
......................................................................aaCauguucuuuauguaggcu..................  3      1  seq
......................................................................aagauguucuGuauguaggcu..................  3      1  seq
......................................................................aagGuguucuuuauguaggcu..................  5      1  seq
......................................................................Gagauguucuuuauguaggcu..................  13     1  seq
......................................................................aagauguucuuuauCuaggcu..................  3      1  seq
......................................................................aagauguAcuuuauguaggcu..................  24     1  seq
......................................................................aagauAucuuuauguaggcu..................  8      1  seq
......................................................................aagauguucuuuauguaCgcu..................  2      1  seq
......................................................................aUgauguucuuuauguaggcu..................  6      1  seq
......................................................................Uagauguucuuuauguaggcu..................  2      1  seq
......................................................................aagauguCcuuuauguaggcu..................  6      1  seq
......................................................................aagauguucuuuaugGaggcu..................  4      1  seq
......................................................................aagauguucuuuaugCaggcu..................  9      1  seq
......................................................................aagUuguucuuuauguaggcu..................  4      1  seq
......................................................................Nagauguucuuuauguaggcu..................  17     1  seq
......................................................................aagauguucuuuauguaUgcu..................  2      1  seq
......................................................................aagauguucuuuaGguaggcu..................  1      1  seq
......................................................................aagauguucuuuaCguaggcu..................  14     1  seq
......................................................................aagauguucuuuauguGggcu..................  6      1  seq
......................................................................aagauguucuuCauguaggcu..................  3      1  seq
......................................................................aagauguucuuuauguagAcu..................  8      1  seq
......................................................................aagauguucuuUuguaggcu..................  6      1  seq
......................................................................aagauNuucuuuauguaggcu..................  1      1  seq
......................................................................aagauguucuuuauguagCcu..................  4      1  seq
......................................................................aagaAguucuuuauguaggcu..................  27     1  seq
......................................................................aagauguucuuGuguaggcu..................  10     1  seq
......................................................................aagaugCucuuuauguaggcu..................  8      1  seq
......................................................................aagauguuAuuuauguaggcu..................  13     1  seq
......................................................................aagauguuGuuuauguaggcu..................  5      1  seq
......................................................................aagauguucuuuauguaggAu..................  29     1  seq
......................................................................aGgauguucuuuauguaggcu..................  4      1  seq
......................................................................aagauguucuCuauguaggcu..................  7      1  seq
......................................................................aagauguucuuuaAguaggcu..................  16     1  seq
......................................................................aagaGguucuuuauguaggcu..................  2      1  seq
......................................................................aagauguucuuuauAuaggcu..................  16     1  seq
......................................................................aagauguucuuuauguUggcu..................  3      1  seq
......................................................................aagauguucuuuauguaAgcu..................  9      1  seq
......................................................................aagauguucuuuauguaggcC..................  89     1  seq
......................................................................aagauguuUuuuauguaggcu..................  14     1  seq
......................................................................aagauguucuuAauguaggcu..................  38     1  seq
......................................................................aagCuguucuuuauguaggcu..................  1      1  seq
......................................................................aaAauguucuuuauguaggcu..................  3      1  seq
......................................................................aagauguucuuuauguaggcA..................  45     1  seq
......................................................................aaUauguucuuuauguaggcu..................  6      1  seq
......................................................................aagauguucuuuauguaggcu..................  60926  0  seq
......................................................................aagauguucuAuauguaggcu..................  14     1  seq
......................................................................aagauguucuuuaugAaggcu..................  31     1  seq
......................................................................aagauguucAuuauguaggcu..................  18     1  seq
......................................................................aagauguucuuuauguagUcu..................  12     1  seq
......................................................................aagauguucuuuauguaAgcuc.................  15     1  seq
......................................................................aagauguucuuuaAguaggcuc.................  12     1  seq
......................................................................aagauguucuuuauguGggcuc.................  11     1  seq
......................................................................aagaCguucuuuauguaggcuc.................  5      1  seq
......................................................................aagUuguucuuuauguaggcuc.................  6      1  seq
......................................................................aagauguucuuuauguaggcuU.................  909    1  seq
......................................................................aagauguucuuuauguaggNuc.................  1      1  seq
......................................................................aagauguucAuuauguaggcuc.................  19     1  seq
......................................................................aaNauguucuuuauguaggcuc.................  1      1  seq
```

Star Mature			
cauuaucauacauucaaaauggcggauuuuagugccuuuauauggaaccaucuauccuuauaucgauuggaagauguucuuuauguaggcuccuguucuucgcccaau			
....Cagauguucuuuauguaggcuc................	1	1	seq
....aGgauguucuuuauguaggcuc................	9	1	seq
....aagauguucCuuauguaggcuc................	12	1	seq
....aagauguucuuuauguaggcuA................	40	1	seq
....aagauUuucuuuauguaggcuc................	3	1	seq
....aagauguucuuuauUuaggcuc................	7	1	seq
....aagauguucuAuauguaggcuc................	25	1	seq
....aagauguucuuCauguaggcuc................	6	1	seq
....aagauguucuuuaCguaggcuc................	9	1	seq
....aagauguucuuuauguaggcuc................	66456	0	seq
....aagauguCcuuuauguaggcuc................	9	1	seq
....aagauguucuuuaugGaggcuc................	2	1	seq
....aagauguucuuuauguaUgcuc................	7	1	seq
....aagauguucuuuGuguaggcuc................	9	1	seq
....aaAauguucuuuauguaggcuc................	8	1	seq
....aagauguGcuuuauguaggcuc................	3	1	seq
....aagauguAcuuuauguaggcuc................	24	1	seq
....aagaAguucuuuauguaggcuc................	37	1	seq
....aagauguucuuuauguUggcuc................	2	1	seq
....aNgauguucuuuauguaggcuc................	2	1	seq
....aagauAuucuuuauguaggcuc................	6	1	seq
....aaUauguucuuuauguaggcuc................	9	1	seq
....aagauguucuuuauguagAcuc................	12	1	seq
....aagauguucuuuauguaggcAc................	12	1	seq
....Nagauguucuuuauguaggcuc................	27	1	seq
....aagauguucuuuauguaggAuc................	29	1	seq
....aagauguucuCuauguaggcuc................	8	1	seq
....aagauguucuuuauguaggcuN................	1	1	seq
....aagauguucuuuaugCaggcuc................	8	1	seq
....aagauguucuuuauguaggGuc................	6	1	seq
....aagauguucuuuauguaggcCc................	12	1	seq
....aagauguucuuuauguaggcuG................	6	1	seq
....Gagauguucuuuauguaggcuc................	8	1	seq
....aagauguuUuuuauguaggcuc................	5	1	seq
....aagauguucuuuauguaggUuc................	9	1	seq
....aagauguucuuuauguagCcuc................	2	1	seq
....aagaugCucuuuauguaggcuc................	7	1	seq
....aagauguucuuuauAuaggcuc................	10	1	seq
....aagaugAucuuuauguaggcuc................	56	1	seq
....aagauguucuuGauguaggcuc................	4	1	seq
....aagauCuucuuuauguaggcuc................	5	1	seq
....aagauguuAuuuauguaggcuc................	8	1	seq
....aagauguucuuuaGguaggcuc................	2	1	seq
....aagaugGucuuuauguaggcuc................	16	1	seq
....aagauguucuuAauguaggcuc................	12	1	seq
....aUgauguucuuuauguaggcuc................	3	1	seq
....aagauguucuuuauguaggcGc................	3	1	seq
....aagauguucGuuauguaggcuc................	6	1	seq
....Uagauguucuuuauguaggcuc................	4	1	seq
....aagauguucuuuaugAaggcuc................	30	1	seq
....aagauguuGuuuauguaggcuc................	8	1	seq
....aagauguucuGuauguaggcuc................	6	1	seq
....aagaGguucuuuauguaggcuc................	5	1	seq
....aagauguucuuuauCuaggcuc................	6	1	seq
....aagauguucuuuauguagUcuc................	2	1	seq
....aagauguucuuuauguaCgcuc................	1	1	seq
....aagGuguucuuuauguaggcuc................	10	1	seq
....aagauguucuuuUuguaggcuc................	7	1	seq
....aagauguucuuuauguUggcucc...............	31	1	seq
....aagauguucuuuaCguaggcucc...............	52	1	seq
....aagaGguucuuuauguaggcucc...............	26	1	seq
....aagauguucuuGauguaggcucc...............	1	1	seq
....aagauguucuuuGuguaggcucc...............	61	1	seq
....aagaugCucuuuauguaggcucc...............	40	1	seq
....aagauguucuuuauUuaggcucc...............	20	1	seq
....aagauguucuAuauguaggcucc...............	133	1	seq
....aagUuguucuuuauguaggcucc...............	28	1	seq
....aagauguucuuuauguaCgcucc...............	11	1	seq
....aagauguucuuuaugAaggcucc...............	198	1	seq
....aagauguuAuuuauguaggcucc...............	55	1	seq

```
                                 Star                                Mature
cauuaucauacauucaaaauggcggauuuuagugccuuuauauggaaccaucuauccuuauaucgauuggaagauguucuuuauguaggcuccuguucuuccgcccaau
......................................................................aagauguucuuuauguaggcuAc................  190  1  seq
......................................................................aagauguucuuuauguaggcuGc................  18  1  seq
......................................................................aagauguucuuuauCuaggcucc................  39  1  seq
......................................................................aagaCguucuuuauguaggcucc................  38  1  seq
......................................................................aagauguucNuuauguaggcucc................  2  1  seq
......................................................................aaCauguucuuuauguaggcucc................  13  1  seq
......................................................................aagauguucAuuauguaggcucc................  133  1  seq
......................................................................aagauguucuuuauguaggGucc................  21  1  seq
......................................................................aGgauguucuuuauguaggcucc................  41  1  seq
......................................................................aagauUucuuuauguaggcucc................  23  1  seq
......................................................................aagauguucuuuauguaggAucc................  133  1  seq
......................................................................aagauguGcuuuauguaggcucc................  9  1  seq
......................................................................aagauguucuuuauguaggcucG................  22  1  seq
......................................................................aagauguCcuuuauguaggcucc................  52  1  seq
......................................................................aagauguucuuuauguGggcucc................  66  1  seq
......................................................................aagauguucuuuauguaggcGcc................  15  1  seq
......................................................................Uagauguucuuuauguaggcucc................  17  1  seq
......................................................................aagauguucuuuaugGaggcucc................  12  1  seq
......................................................................Nagauguucuuuauguaggcucc................  113  1  seq
......................................................................aagauguucuuuauguagCcucc................  13  1  seq
......................................................................aagauguucuuuauguaggcuNc................  1  1  seq
......................................................................aagauguucuuuaNguaggcucc................  1  1  seq
......................................................................aagauguucuuuaGguaggcucc................  17  1  seq
......................................................................aagauguucuuAauguaggcucc................  84  1  seq
......................................................................aagauguucuuuauAuaggcucc................  57  1  seq
......................................................................aUgauguucuuuauguaggcucc................  25  1  seq
......................................................................aCgauguucuuuauguaggcucc................  2  1  seq
......................................................................aagauNuucuuuauguaggcucc................  3  1  seq
......................................................................aagauguucuuuauguagAcucc................  60  1  seq
......................................................................aagauguucuuuauguaggcucU................  1904  1  seq
......................................................................aagauguucuuuauguaggcucN................  3  1  seq
......................................................................aaNauguucuuuauguaggcucc................  1  1  seq
......................................................................aagauguucuuuauguaggcCcc................  46  1  seq
......................................................................aagaugGucuuuauguaggcucc................  64  1  seq
......................................................................aagaAguucuuuauguaggcucc................  157  1  seq
......................................................................aagauguucuuuauguaggcucc................  363982  0  seq
......................................................................Gagauguucuuuauguaggcucc................  104  1  seq
......................................................................aagauguucuuuauguaggUucc................  21  1  seq
......................................................................aagauAuucuuuauguaggcucc................  43  1  seq
......................................................................aagauguAcuuuauguaggcucc................  103  1  seq
......................................................................aagauguucGuuauguaggcucc................  37  1  seq
......................................................................aagauguucuuuauguaggcuUc................  183  1  seq
......................................................................aagauguucuuuaAguaggcucc................  89  1  seq
......................................................................aagaugAucuuuauguaggcucc................  288  1  seq
......................................................................aagauguucuuuCuguaggcucc................  3  1  seq
......................................................................aagCuguucuuuauguaggcucc................  2  1  seq
......................................................................aaUauguucuuuauguaggcucc................  58  1  seq
......................................................................aagauCuucuuuauguaggcucc................  20  1  seq
......................................................................aagauguucCuuauguaggcucc................  98  1  seq
......................................................................aagauguucuGuauguaggcucc................  17  1  seq
......................................................................aagGuguucuuuauguaggcucc................  46  1  seq
......................................................................aagauguucuuuauguaAgcucc................  90  1  seq
......................................................................aagauguucuuuauguaggcAcc................  90  1  seq
......................................................................aagauguucuuuUuguaggcucc................  34  1  seq
......................................................................aagauguuUuuuauguaggcucc................  90  1  seq
......................................................................aagauguucuuuauguaUgcucc................  113  1  seq
......................................................................aagauguucuuCauguaggcucc................  40  1  seq
......................................................................aagauguucuuuauguCggcucc................  2  1  seq
......................................................................aagauguucuuuaugNaggcucc................  1  1  seq
......................................................................aagauguuGuuuauguaggcucc................  27  1  seq
......................................................................aaAauguucuuuauguaggcucc................  35  1  seq
......................................................................aagauguucuuuaugCaggcucc................  45  1  seq
......................................................................aNgauguucuuuauguaggcucc................  26  1  seq
......................................................................aagauguucuCuauguaggcucc................  46  1  seq
......................................................................aagauguucuuuauguagUcucc................  15  1  seq
......................................................................aagauguucuuuauguaggcucA................  348  1  seq
......................................................................Cagauguucuuuauguaggcucc................  2  1  seq
......................................................................aagaugAucuuuauguaggcuccu...............  3  1  seq
......................................................................aagauguucuuuauguaggcucAu...............  9  1  seq
......................................................................aagauguAcuuuauguaggcuccu...............  2  1  seq
```

```
                                Star                                Mature
cauuaucauacauucaaaauggcggauuuuagugccuuuauauggaaccaucuaucc uuauaucgauuggaagauguucuuuauguaggcuccuguucuuccgcccaau
......................................................................aagUuguucuuuauguaggcuccu...............    1      1     seq
......................................................................aUgauguucuuuauguaggcuccu...............    1      1     seq
......................................................................aagauguucuAuauguaggcuccu...............    2      1     seq
......................................................................aagauguucuuuauguaggcuGcu...............    1      1     seq
......................................................................aagauguucuuuauguaggcuAcu...............    2      1     seq
......................................................................aNgauguucuuuauguaggcuccu...............    1      1     seq
......................................................................aagauguCcuuuauguaggcuccu...............    1      1     seq
......................................................................aagauguucCuuauguaggcuccu...............    2      1     seq
......................................................................aagauguucuuAauguaggcuccu...............    1      1     seq
......................................................................aagauguucuuuauguaggcAccu...............    4      1     seq
......................................................................aagauguucuuuauguaggcuccG...............    20     1     seq
......................................................................aagauguucuuuauguaggcuccC...............    78     1     seq
......................................................................Uagauguucuuuauguaggcuccu...............    2      1     seq
......................................................................aagaCguucuuuauguaggcuccu...............    1      1     seq
......................................................................aagauguucuuuGuguaggcuccu...............    1      1     seq
......................................................................aagauguucuuuauguUggcuccu...............    1      1     seq
......................................................................aagauguucuuuauguaggcuUcu...............    2      1     seq
......................................................................aagaugCucuuuauguaggcuccu...............    2      1     seq
......................................................................aagauguucuuuaCguaggcuccu...............    3      1     seq
......................................................................aagaAguucuuuauguaggcuccu...............    2      1     seq
......................................................................aagauguucuuuaugCaggcuccu...............    2      1     seq
......................................................................aGgauguucuuuauguaggcuccu...............    2      1     seq
......................................................................aagauguucuuuauguaggcuccu...............    8055   0     seq
......................................................................aagGuguucuuuauguaggcuccu...............    1      1     seq
......................................................................aagauguucuuuauguaggcCccu...............    2      1     seq
......................................................................aagauUuucuuuauguaggcuccu...............    1      1     seq
......................................................................aagauguucuuuauguaggcuccA...............    673    1     seq
......................................................................aagauguucuuuauguagCcuccu...............    1      1     seq
......................................................................aagauguucuuuauguagAcuccu...............    1      1     seq
......................................................................Nagauguucuuuauguaggcuccu...............    1      1     seq
......................................................................aagauguucuuuauCuaggcuccu...............    1      1     seq
......................................................................aagauguucAuuauguaggcuccu...............    2      1     seq
......................................................................aagaugGucuuuauguaggcuccu...............    3      1     seq
......................................................................Gagauguucuuuauguaggcuccu...............    1      1     seq
......................................................................aagauguucuuuauguaggcucUu...............    15     1     seq
......................................................................aagauguucuuuauguaggAuccu...............    4      1     seq
......................................................................aagauguucuuuauguagUcuccu...............    1      1     seq
......................................................................aagauguucuuuauguaUgcuccu...............    1      1     seq
......................................................................aagauguucuuuauguaggcuccuA..............    8      1     seq
......................................................................aagauguucuuuauguaggcuccuU..............    383    1     seq
......................................................................aagauguucuuuauguaggcuccuAu.............    33     1     seq
......................................................................aagauguucuuuauguaggcuccuUu.............    68     1     seq
......................................................................aagauguucuuuauguaggcuccuCu.............    22     1     seq
......................................................................aagauguucuuuauguaggcuccuAuu............    21     1     seq
......................................................................aagauguucuuuauguaggcuccuUuu............    14     1     seq
.......................................................................agauguucCuuauguaggcuc.................    1      1     seq
.......................................................................agauguucuuuauguaggcuc.................    2      0     seq
.......................................................................agauguucuuuauguaggcucc................    22     0     seq
.......................................................................aAauguucuuuauguaggcucc................    1      1     seq
.......................................................................aUauguucuuuauguaggcucc................    2      1     seq
.......................................................................Ugauguucuuuauguaggcucc................    1      1     seq
.......................................................................agauguucuuuauguaggcuccu...............    3      0     seq
........................................................................gauguucuuuauguaggcu..................    2      0     seq
........................................................................Aauguucuuuauguaggcucc................    2      1     seq
........................................................................gauguucuuuauguaggcucc................    111    0     seq
........................................................................gauguucuuuauguaggcucU................    1      1     seq
.........................................................................auguucuuuauguaggcu..................    3      0     seq
.........................................................................auguucuuuauguaggcuc.................    1      0     seq
.........................................................................auguucuuuauguaggcucc................    29     0     seq
.........................................................................auguucuuuauguaggcuccu...............    275    0     seq
.........................................................................auguucuAuauguaggcuccu...............    1      1     seq
.........................................................................auguucuuuauguaggcuccA...............    9      1     seq
.........................................................................auguucuuuauguaggcuccuA..............    4      1     seq
.........................................................................auguucuuuauguaggcuccuU..............    1      1     seq
..........................................................................uguucuuuauguaggcucc................    16     0     seq
..........................................................................uguucuuuauguaggcuccu...............    1      0     seq
...........................................................................guucuuuauguaggcucc................    5      0     seq
```
